# Methicillin-resistant *Staphylococcus aureus *in the ICU: risk factors and a predictive model to detect it at ICU admission

**DOI:** 10.1186/cc14181

**Published:** 2015-03-16

**Authors:** F Callejo-Torre, JM Eiros, S Ossa-Echeverri, P Olaechea, M Palomar, F Alvarez-Lerma, Envin-Helics Study Group

**Affiliations:** 1 Hospital Universitario de Burgos, Spain; 2 Hospital Clínico Universitario de Valladolid, Spain; 3 Hospital de Galdakao-Usansolo, Galdakao, Spain; 4 Hospital Arnau de Villanova, Lleida, Spain; 5 Hospital del Mar, Barcelona, Spain

## Introduction

Being capable of predicting MRSA on ICU admission is crucial to enhance infection control and to avoid inappropriate empirical treatment. Two objectives were studied: to describe risk factors for MRSA colonization/infection (MRSA-C/I) once admitted to the ICU; and to develop a predictive model at ICU admission, based on easy-to-obtain admission factors.

## Methods

Data were collected prospectively from 69,894 patients admitted consecutively (stay >24 hours) to 147 Spanish ICUs participating in the National Surveillance Study of Nosocomial Infections in ICU registry (ENVIN) during April-to June 2006 to 2010. Univariable and multivariable analysis was performed for both objectives but we used only easy-to-obtain variables for the predictive model exclusively from those admitted in 2010 (*n *= 16,950, 2/3 for analysis and 1/3 for subsequent validation).

## Results

In the 2006 to 2010 period, 1,046 were C/I by MRSA (note that relative risks are not included due to space limitations). First objective: previous antibiotic, APACHE II score >18, skin-soft tissue or postsurgical superficial skin infections, trauma or medical patient, age >65 (especially >75), urinary catheter and admitted from a long-term care facility were independent risk factors for MRSA-C/I in ICU. Multicolonization increased significantly the risk of MRSA-C/I, and immunodeficiency and gender male emerged as protective factors. Second objective: independent risk factors on ICU admission were male gender, trauma critical patient, urgent surgery, admitted from other ICU, community or long-term facility, being immunosuppressed and skin-soft tissue infection. All configured the risk model for which, although showing good discrimination (AUC-ROC, 0.77; 95% CI, 0.72 to 0.82), sensitivity (67%) and specificity (76.5%) were insufficient for the ICU setting. Afterwards validation with the remaining 4,952 (1/3) showed AUC-ROC = 0.72 (95% CI, 0.65 to 0.79) and *P *value on the Hosmer-Lemeshow goodness of fit test = 0.539. The model did not improve even after including more complex variables (AUC-ROC = 0.82; 95% CI, 0.77 to 0.86, sensitivity 63.64%, specificity 78.48%).

## Conclusion

Independent risk factors for MRSA-C/I in the ICU and at ICU admission are described. To predict MRSA-C/I at ICU admission we should not rely on clinical-demographic risk factors alone. Its combination with a rapid laboratory test could be the way to proceed in future studies.

